# Supplementary material for: Fin Cells as a Promising Seed Cell Source for Sustainable Fish Meat Cultivation
Source: Foods. 2025 Jun 12;14(12):2075. doi: 10.3390/foods14122075 (PMC12191693; doi:10.3390/foods14122075)
Supplement: Supplementary file 1 [file foods-14-02075-s001.zip › foods-3623674-supplementary.pdf]

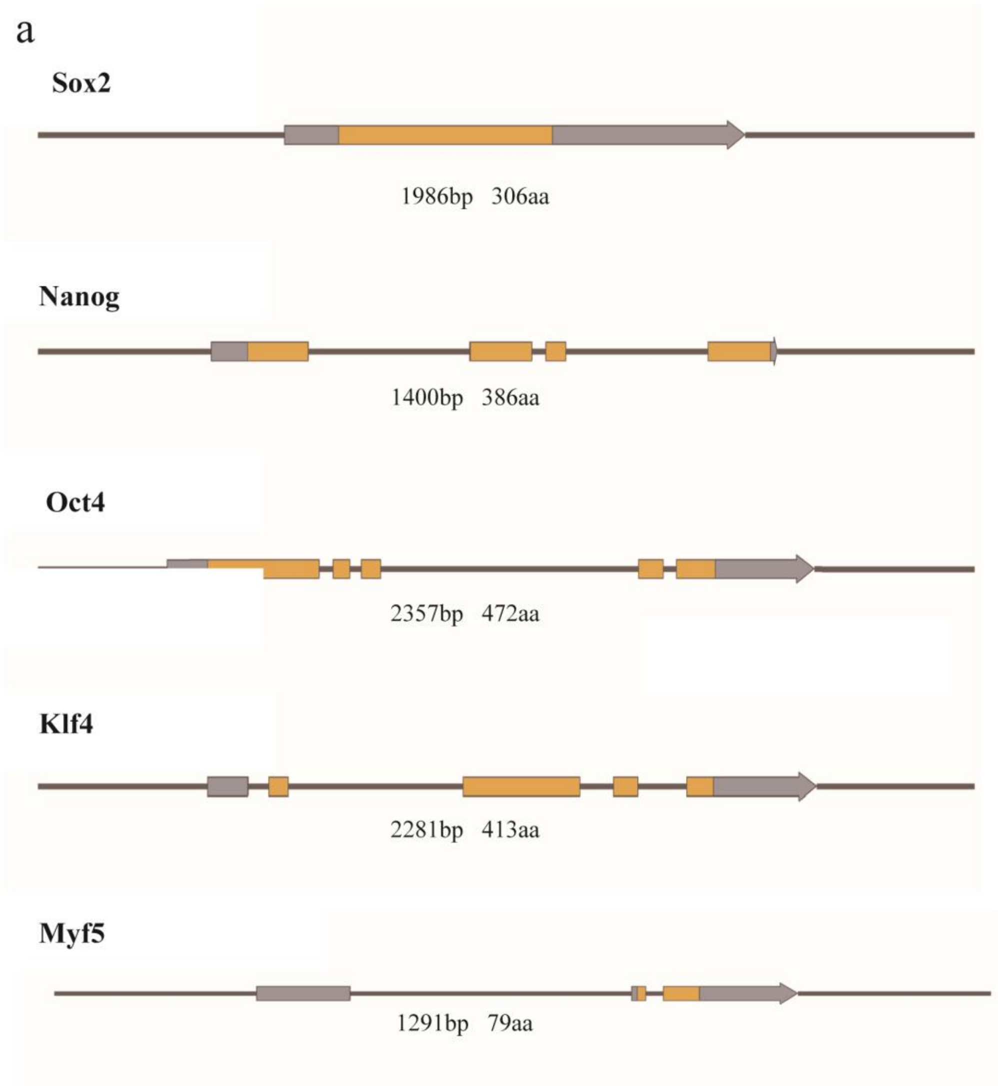

Figure. S1: Gene structure prediction

a. Gene structure of Culter alburnus. The gray box is the UTR sequences, and the yellow box is the CDS sequences.

Table S1 Primer of RT-PCR

Name of primer	Sequence(5' to 3')		Target
Sox2	F	GAACGCCTTCATGGTGTGGTC	128
	R	GCTTCTCGCTCTCGGACAGA	
Nanog	F	ATCTCCAGCCCGACCTCCAA	133
	R	TCCAGCAAGCGTCTTCATCTCT	
Oct4	F	CCGTTCTGCTCTGGAGTCATAC	174
	R	ACTTCGGCACATTCGTCATCAA	
c-Myc	F	TCCCTCTGTGGTCTTCCCTTAT	220
	R	CTTCTCCACTGTCACCACATCA	
Klf4	F	ATGACCAAGAAGGAGCCAGAAG	167
	R	GGCATCTGATAGGTGTAGGACG	
β-actin	F	GACAGGTCATCACCATTGGCAAT	157
	R	GTGTTGGCATACAGGTCCTTACG	

Table S2 Primer of RT-qPCR

Name of primer	Sequence(5' to 3')		Target
Myf5	F	CCAGGAGTGTCTAGTTTGCAGT	132
	R	AGAACTGCACTGGGAATCACTT	
Myha	F	CTGGCTTGTCCTGCGTCACT	136
	R	TGGTAGGCGTTGTCGGAGATG	
